# Computational modelling unravels the precise clockwork of cyanobacteria

**DOI:** 10.1098/rsfs.2018.0038

**Published:** 2018-10-19

**Authors:** Nicolas M. Schmelling, Ilka M. Axmann

**Affiliations:** Institute for Synthetic Microbiology, Cluster of Excellence on Plant Sciences (CEPLAS), Heinrich Heine University Düsseldorf, Universitätsstraße 1, Düsseldorf 40225, Germany

**Keywords:** circadian clock, oscillation, entrainment, adaptation, noise, robustness

## Abstract

Precisely timing the regulation of gene expression by anticipating recurring environmental changes is a fundamental part of global gene regulation. Circadian clocks are one form of this regulation, which is found in both eukaryotes and prokaryotes, providing a fitness advantage for these organisms. Whereas many different eukaryotic groups harbour circadian clocks, cyanobacteria are the only known oxygenic phototrophic prokaryotes to regulate large parts of their genes in a circadian fashion. A decade of intensive research on the mechanisms and functionality using computational and mathematical approaches in addition to the detailed biochemical and biophysical understanding make this the best understood circadian clock. Here, we summarize the findings and insights into various parts of the cyanobacterial circadian clock made by mathematical modelling. These findings have implications for eukaryotic circadian research as well as synthetic biology harnessing the power and efficiency of global gene regulation.

## 1. Introduction

Life on Earth has evolved under the influence of changing environmental conditions. While some environmental conditions may fluctuate without any apparent regularity, a large class of environmental changes exhibits regular cycles on daily to annual time scales. The most prominent of these environmental cycles is the daily change in temperature and light availability caused by Earth’s rotation itself. Circadian clocks are an adaptation to this recurring cycle.

Circadian clocks are biochemical oscillators that function as an endogenous timekeeper within organisms. Circadian clocks are characterized by three properties: (i) persistence of oscillations without an exogenous cycle, e.g. in constant light, (ii) temperature compensation and (iii) the entrainment of the endogenous oscillator(s) to the exogenous cycle of light and darkness [1]. Without the exogenous stimulus (Zeitgeber) the period length of the oscillator can vary between organisms. The ability of entrainment, however, allows the Zeitgeber to synchronize the oscillation with the exogenous rhythm.

While changing environmental conditions typically pose a challenge to organisms, changing conditions also present the possibility of adaptation and therefore a fitness advantage over competitors. The fact that circadian clocks can be found in a variety of organisms from mammals to plants and fungi suggests a selective pressure by environmental light/dark cycles that favoured the development of such a complex timing system. A fitness advantage of circadian clocks has been elegantly shown in competition experiments using cyanobacteria with intact circadian clocks and clock mutants [2,3]. Circadian clocks appear to be a conserved trait in evolution. However, differences in the sequences of proteins that are involved in the circadian clock suggest a convergent evolution of timing mechanisms [4,5].

The history of research on the genetic basis of circadian clocks begins in the 1970s when Konopka and Benzer first identified clock components in *Drosophila melanogaster* [6]. Afterwards, circadian clocks were also identified in mammals, including hamsters, mice and humans [7–9], as well as fungi with *Neurospora crassa* [10] and plants with *Arabidopsis thaliana* [11]. In the beginning, it was believed that circadian clocks were exclusive to eukaryotic organisms and that simpler prokaryotic organisms could simply not maintain independent circadian oscillation as they divide multiple times during a day [1]. However, in the mid-1980s, researchers identified oscillations in different diazotrophic cyanobacteria, performing photosynthesis during the day and fixing nitrogen at night [1]. At least since the remarkable findings of Kondo and his colleagues in 1993 identifying circadian gene expression in cyanobacteria and later the central three proteins of the cyanobacterial circadian clock KaiA, KaiB and KaiC, all doubts that simple prokaryotic organisms can harbour such complex systems as circadian clocks were removed [12,13].

In this review, we will cover various computational concepts of circadian clocks. We will first introduce basic functionality and the differences between eukaryotic and cyanobacterial circadian clocks. Afterward, we will focus on the cyanobacterial circadian clock system as this is the evolutionary oldest known yet functional clock system. Furthermore, the cyanobacterial clock is special in the sense that it does not rely on transcriptional processes and can stably oscillate in a test tube for weeks. In addition, over the last decades central processes and characteristics of circadian clocks have been studied experimentally and computationally, including entrainment strategies, the adaptation to noise and environmental changes, as well as robustness concepts, using the cyanobacterial system. Further aspects and information about computing by biological clocks, which are not covered in this review, can be found in a recent review by Dalchau *et al*. [14].

## 2. Basic functionality of circadian clocks

Circadian clocks are a prominent way to coordinate and regulate gene expression on a global cell scale. Instead of regulating single genes or an operon, circadian clocks are able to regulate hundreds to thousands of genes. In *Synechococcus elongatus* PCC 7942, the model organism for cyanobacterial circadian clock research, up to 64% of the genome is under the control of the circadian clock [15]. Other cyanobacteria also show oscillations of large portion of their genomes ranging 20–79% [16] compared with eukaryotic organisms, which show only 5–15% of rhythmically oscillating transcripts [17].

However, not everything that oscillates is under the control of a circadian clock. As mentioned above, circadian clocks are characterized by three essential criteria [1]. In *Saccharomyces cerevisiae*, even under constant optimal growth conditions, global gene expression oscillates. So far no circadian clock has been identified in yeast and these oscillations are a result of a rather general growth principle where changes in genome configuration are due to changes in the energy levels of the cell [18,19].

Even though there are phenomena which cause stable oscillations in global gene expression, e.g. supercoiling of the DNA, circadian clocks are a prevalent form found in almost any group of organisms. It has been hypothesized that the cyanobacterial clock, however, is influencing the supercoiling state of the DNA thereby connecting the circadian system’s output with a global gene expression system [15,17]. Circadian clocks are divided into two groups in regard to their functionality: Eukaryotic circadian clocks comprise nested transcription–translation feedback loops (TTFL), whereas cyanobacterial circadian clocks, as the only known example for prokaryotic circadian clocks, comprise a post-translational oscillator (PTO) [5]. As mentioned before, sequence analyses suggest a convergent evolution with multiple origins of circadian clocks, which is supported by the different functionality of known circadian clocks.

Even though eukaryotic circadian clocks do not share sequence similarities between the protein factors involved, they share structural similar circuits as all of them comprise nested positive and negative gene–protein feedback loops. However, based on the activation or inhibition of gene expression of clock factors, TTFL clocks are susceptible to effects of DNA replication or gene position on the chromosome as we will discuss later.

**Figure 1.**
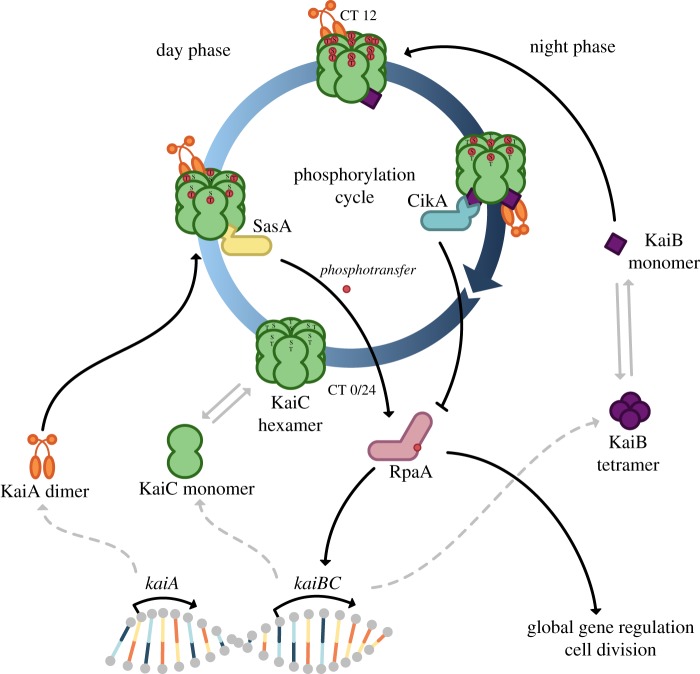
Circadian clock of *Synechococcus elongatus* PCC 7942 and its interaction network. The interactions of the core clock result in a 24 h cycle of phosphorylation and dephosphorylation. Depending on the phosphorylation state of KaiC hexamers different proteins interact with the core clock to connect the circadian signal to global gene regulation. SasA and CikA comprise an antagonistic system, which regulates the activity of RpaA and thus its ability to bind promoters. The figure is adapted from [16,20,21]. Graphical representations of the protein factors involved are based on ‘Cyanobacterial Circadian Clock Output Mechanism’ by The BioClock Studio (https://youtu.be/DcuKifCRx_k). The colouring of the Kai proteins is adapted to the colouring of the protein structures of the Kai proteins from [22]. Dashed lines represent transcription and translation processes. Solid lines represent physical interactions between proteins. Grey solid lines represent de-/formations of homo-multimers.

In cyanobacteria, the circadian clock comprises three proteins KaiA, KaiB and KaiC. At the centre of the cyanobacterial circadian clock is a phosphorylation cycle. In detail, cyanobacteria measure time by the phosphorylation state of KaiC, which is the central part of the clock. KaiC auto-phosphorylates during the day stimulated by KaiA (figure 1).

At dusk, KaiC is completely phosphorylated and a conformational change allows KaiB to bind to KaiC. KaiB then antagonizes the positive effect of KaiA and initiates auto-dephosphorylation by sequestering KaiA. At dawn, KaiC is completely dephosphorylated, which causes KaiB to be no longer able to bind to KaiC, the KaiCBA complex falls apart and the cycle starts all over again with free KaiA binding to KaiC and stimulating the phosphorylation [22,23].

After reconstructing the phosphorylation mechanism of KaiC in a test tube [24], multiple computational models using differential equations arose in order to elucidate the functionality of the cyanobacterial circadian clock. It was proposed that shuffling and exchange of KaiC monomers between KaiC hexamer is important for stability and synchronicity of the oscillation [25–27]. Further, the remarkably robust high-amplitude phosphorylation cycles of KaiC are achieved by sequestration of free KaiA mediating synchronization [5,28–31]. This sequestration mechanism is a conserved design principle in natural systems, which can also be found in many different systems, e.g. MAPK protein kinase cascade [32,33]. Shortly after, other models resolved the importance of KaiA and KaiB in the cyclic phosphorylation of KaiC [31,34–36]. Each KaiC monomer has two independent phosphorylation sites, and it could experimentally be shown by Rust and colleagues that there is an ordered phosphorylation of these sites, which was before neglected by other models [37]. Further, their model showed that KaiA is inactivated when the concentration of serine phosphorylated KaiC exceeds a certain threshold. Shortly after Brettschneider and colleagues built an advanced model on top of the model proposed by Rust and co-workers, which showed for the first time the ability of entrainment by temperature [28]. This mathematical model by Brettschneider *et al*. [28] suggested that the complex of KaiCpS/T and KaiB binds KaiA dimers at a newly formed binding site thereby inhibiting KaiA-mediated stimulation of KaiC phosphorylation, which was confirmed just recently by single-particle cryo-electron microscopy and mass spectrometry [22,23].

Computational models have helped to resolve open questions about the dynamics of interactions between clock factors in cyanobacteria. However, in eukaryotic circadian clocks protein factors regularly activate or suppress gene expression of their own expression or of other clock factors [38]. In cyanobacteria, a similar negative feedback mechanism was suggested by Iwasaki *et al*. [39] in which phosphorylated KaiC inhibits the expression of the *kaiBC* mRNA. Computational models could first confirm those suggestions by showing that such a negative feedback mechanism can, in fact, explain sustained oscillation in *kaiBC* expression [40]. Later, Hertel *et al*. [20] could also identify which of the four phosphorylation states of KaiC suppresses *kaiBC* expression and that even two forms activate its own expression.

The circadian clock of the model organism *Synechococcus elongatus* PCC 7942 was the basis for all of the above-described models. However, cyanobacteria are characteristic for their diversity, which can also be observed regarding the composition of circadian clock factors. With respect to this composition, three distinct groups of cyanobacteria can be identified: (i) *Synechococcus*-like clock composition harbouring single copies of each of the known factors in the circadian clock of *Synechococcus elongatus* PCC 7942, (ii) a *Synechocystis*-like clock composition harbouring multiple copies of some factors including KaiB and KaiC and (iii) a *Prochlorococcus*-like clock, which is missing multiple factors of input and output pathways as well as the central factor KaiA [16,41,42]. Interestingly, the *kaiA* gene never occurs more than once in a genome and cannot be found outside cyanobacteria in contrast to other core clock factors. Furthermore, the *Prochlorococcus* clock is called an hourglass timing system and is not considered a circadian clock as it does not persist without an exogenous cycle but shows oscillation of global gene expression under a light–dark regime [43]. It remains an open question how the multiple copies of the core clock factors in *Synechocystis* are integrated into the circadian clock.

## 3. Entrainment

One defining characteristic of a circadian clock is the ability to be entrained to an exogenous cycle. It is important that a circadian system can be synchronized to an exogenous stimulus in order to be most useful for the organism in anticipating recurring patterns. However, it is also necessary that such a system is robust against naturally occurring fluctuation of the input signal.

There are two strategies for entraining the clock to an exogenous stimulus: (i) with a direct sensing of light intensity by clock components or other components that pass the signal onto clock components, which strategy is normally used by eukaryotic circadian clock systems [4] and (ii) indirect sensing of light through changes in the metabolic state of the cell, i.e. redox state or adenosine triphosphate (ATP)/adenosine diphosphate (ADP) ratio, which is commonly used by cyanobacteria [21,44]. The core oscillator of the cyanobacterial circadian clock depends as above described on the phosphorylation state of KaiC. KaiC is able to autophosphorylate and -dephosphorylate, which is enhanced by the interaction with the other core factors KaiA and KaiB. Each monomer has two phosphorylation sites, which in the end results in 12 independent sites for each hexamer. The phosphorylation pattern of KaiC hexamers is highly ordered resulting in cycles of the following four states: unphosphorylated (U-KaiC), phosphorylated only on S431 (S-KaiC), phosphorylated only on T432 (T-KaiC) and phosphorylated on both S431 and T432 (ST-KaiC) [37]. It has experimentally been shown that the phase of the circadian clock is affected by the ATP/ADP ratio, which is a result of the cellular catabolic metabolism and the photosynthetic apparatus [21]. Dark phases cause a drop in the ATP/ADP ratio, which shifts the clock into the dephosphorylation phase. Rust *et al*. [44] convincingly showed that the *in vitro* form of the cyanobacterial circadian clock reacts differently to the ATP/ADP ratio depending on the timing of the dark phase. During the phosphorylation phase (subjective day), the oscillator is most susceptible to changes in the ATP/ADP ratio, whereas in the dephosphorylation phase (subjective night) the oscillator is almost insensitive. Adding the competitive inhibitor to their core clock model, they described an entrainment mechanism for the cyanobacterial circadian clock that works *in vitro* and does not rely on an additional signalling pathway, resembling the direct effect of the metabolic state of the cell on the phase of the circadian clock [44].

Such an entrainment strategy seems like a very early form as it does not rely on other protein factors or special compounds. ATP and ADP are most likely older than the KaiC or its ancestral version, meaning that the core of a clock comprised of a single protein, KaiC or its ancestor, and the cellular energy compound ATP and its derivatives could have arisen early in evolution. A recent mathematical model by Hernansaiz-Ballesteros *et al*. [45] demonstrated how a single molecule similar to KaiC can form an oscillator through ordered phosphorylation. The model was based on a two-intermediate system as an alteration of an ‘approximate majority’ algorithm and systematically removed reaction paths between the different phosphorylation states. In the end, the model predicted a system with similar ordered reaction paths as seen for KaiC [45]. Thus, KaiC could have evolved as an ATP biosensor and ancient clock and later more protein factors were added to the clock in order to make it more robust to external and intrinsic fluctuations as well as to create a more robust readout. This is in good agreement with sequencing analyses, which suggest that KaiC originated first from a RecA (bacterial DNA recombination protein) ancestor by gene duplication and subsequent fusion before KaiB and last KaiA originated [41]. Other protein factors of the circadian clock are evolutionarily younger or as old as KaiB. For example, SasA, the first downstream interaction partner of KaiC (figure 1), is thought to have originated from a fusion of a two-component histidine kinase and an ancestor of KaiB around the time when KaiB formed a cluster with KaiC [46].

However, what are the environmental conditions that led to the evolution of circadian clocks? What conditions would favour the evolution of such a complex system and provide a fitness advantage for the organism? Troein *et al*. [47] attempted to answer those questions by evolving *in silico* gene regulatory networks to best predict phases of a day/night cycle. Those gene regulatory networks best resemble the eukaryotic circadian clock systems; however, their findings can still be considered to be universally correct for other circadian clock systems. Troein and co-workers showed that only under multiple photoperiods combined with environmental noise were circadian clocks able to evolve with a high probability. In addition, real environmental variations even increase this probability. They concluded that only seasonally changing photoperiods combined with sufficient noise can lead to the evolution of circadian clocks, which is accompanied by an increase in complexity of the system [47].

It has been shown through sequence analyses that cyanobacterial clocks are evolutionarily old systems and by mathematical modelling that seasons and noise are needed to promote the evolution of such a system. Nevertheless, one aspect of the evolution of circadian clocks is still missing. All above-mentioned simulations assume a day length of roughly 24 h; however, the length of day changed drastically over the last 2.5 billion years and thus also during the evolution of circadian clocks [48]. Based on measurements of sandstone laminae, the length of a solar day on Earth is estimated to be 17 h [48]. Furthermore, models about the origin of the Moon 4.47 billion years ago predict a rotational speed of the Earth between 2.5 and 9 h [49–51]. How does the length of day affect the complexity of circadian clocks? Were additional factors also needed to adjust the biochemical speed of circadian clocks in addition to increase robustness?

## 4. Adaptation of circadian clocks to noise and environmental changes

As described before, noise and environmental changes, e.g. seasonal changes in the photoperiod, are essential for the evolution of circadian clocks [47]. The importance of noise for the evolution of circadian clocks also highlights the potential for adaptation. Circadian clocks are one of the naturally occurring adaptations to recurring noisy patterns, e.g. noisy light–dark cycles. They provide a fitness advantage to organisms by robustly buffering such noise. Further, circadian clocks are able to adjust the oscillator’s period to long-term seasonal changes in the environment. As Troein *et al*. [47] showed, one strategy of adaptation is to increase the number of feedback loops and complexity of gene regulatory networks. However, the cyanobacterial circadian clock has a PTO at the centre, which functions in a different manner from eukaryotic TTFLs. In addition, there are two forms of timing mechanisms in cyanobacteria: (i) a circadian clock as seen in *Synechococcus*, which functions similar to a limit cycle oscillator [52,53] and (ii) an hourglass timing system as seen in *Prochlorococcus*, which works more like a point attractor that stops oscillating and relaxes to a stable fixed point in the absence of an exogenous cycle [53]. Interestingly, recent findings of microbial communities with *Prochlorococcus* indicate prolonged oscillations of transcripts in extended darkness [54]. These findings suggest a more complex exogenous network in addition to the light/dark cycle to entrain the timing system in nature. The interactions in the coculture seem to cause changes of the redox state in *Prochlorococcus* cells, which are similar to the ones found under a light/dark regime and thus prolong the oscillations [54].

How do these two systems cope with noise? Before answering this question we first have to distinguish between external noise by variations in the exogenous stimulus, e.g. light availability, and internal noise due to variations in biochemical reactions. External noise such as shading by clouds can have drastic effects on the availability of light. Such fluctuations can also range in their time scale from seconds to hours, which can cause the circadian clock to be shifted back to night cycles [55]. Fluctuations of external cues have different effects on the two circadian clock systems in cyanobacteria. Whereas, the limit cycle oscillator such as the circadian clock from *Synechococcus* is almost unaffected by such fluctuations [53,56], the point attractor like the hourglass timing system from *Prochlorococcus* is set in free fall towards the night state [53]. The ability to tell the accurate time increases with the size of the limit cycle and the overlap between the day and night state of the system [53]. On the other side, when considering only internal noise due to finite numbers of proteins the point attractor outperforms the limit cycle in regard to precision due to its ability to change faster between both states [53].

Limit cycle oscillators are more robust under a range of external and even internal noise levels; however, so far only noise in a 12 L : 12 D regime was analysed. As previously described, in addition to noise also seasonal changes are required for the evolution of circadian clocks [47]. How do these circadian clocks adapt to seasonal changes in the day length? To answer this question, Leypunskiy and colleagues analysed the effects of different day length on the performance of the circadian clock of *Synechococcus elongatus* PCC 7942 *in vitro* and *in vivo*. Interestingly, they could show that after a transient phase the circadian clock tracks midday over a large range of day lengths ranging from 6 to 20 h of light. This observation is also found in the *in vitro* clock in a test tube, where similar to light and dark phases, phases of high ATP or high ADP are alternating, resembling the intercellular effect of light on the levels of ATP and ADP through photosynthesis [57]. The effects of seasonality and different photoperiods on entrainment are studied theoretically in further detail by Schmal *et al*. [58].

The cyanobacterial circadian clock has the ability to perform under various conditions regarding the length of day and the level of external and internal noise; however, the molecular mechanisms of how the circadian clock is able to cope with these variations remain unknown. By comparing three models that try to explain some of the molecular mechanisms of input compensation Paijmans *et al*. [56] were able to identify a new mechanism at the individual hexamer level. Whereas the period of the circadian clock in their model [59] remains unaffected by changes in the bulk ATP fraction, the models by Rust *et al*. [37] and van Zon *et al*. [31] show decreasing or increasing period lengths by decreasing ATP amount, respectively. This high level of input compensation of the Paijmans model is achieved on the individual hexamer level rather the population level. At increasing ATP levels, the phosphorylation rate increases, which is true for all models, resulting in an increased cycle length of each hexamer through the phosphorylation cycle. Meaning that under decreasing ATP levels, input compensation is achieved through smaller cycles in the phosphorylation state space by individual hexamers and an earlier switching to the inactive state of these hexamers [56]. This mechanism provides a way to explain the robustness of this circadian clock system to external noise by changes in the light availability and a subsequent drop in the ATP levels.

Even though the circadian clock shows great robustness to noise and unexpected changes in the availability of energy through sunlight, it is still vulnerable to large drops in ATP levels at certain times of day. Looking at the phase response curves for the three previously mentioned models, we see that early in the day a dark pulse has the strongest effect on the clock by delaying its phase [56]. In order to analyse the cost associated with this vulnerability, Lambert *et al*. [55] studied the effects of misalignment of the circadian clock to the environment in individual cyanobacterial cells [55]. Early in the day cells are most vulnerable to sudden drops in ATP levels resulting in a growth arrest, which can lead to a failure to resume to growth when cells are placed into the light. At the beginning of the subjective day, the cells are rapidly growing, whereas during subjective night, especially at dusk, the cells are most resistant against starvation, which potentially corresponds to the internal glycogen storage [55]. Thus, the authors conclude that one of the major functions of the cyanobacterial circadian clock is to balance between rapid growth and starvation resistance.

## 5. Further strategies for robustness of a circadian clock

As highlighted before, one of the most important tasks of a circadian clock is the ability to robustly measure and transfer an exogenous signal to downstream processes. We have seen that noise is essential for the evolution of the circadian clock and that the cyanobacterial clock has evolved different strategies to cope with external and internal noise in order to robustly predict the period of the exogenous cycle. Furthermore, it was shown that independent of the length of the exogenous stimulus, i.e. light, the cyanobacterial circadian clock tracks the middle of that phase in order to predict to the upcoming change.

There are, however, further strategies for robustness of circadian clocks and oscillators. Some of which are already found in the circadian clock of cyanobacteria, others are strategies that can be applied in order to make synthetic oscillators more robust.

Eukaryotic circadian clocks comprise nested TTFLs where gene products regulate the expression of other factors of the clock. One common motif in these TTFLs is the self-repression of the gene by the gene product, which is found in prokaryotic as well as eukaryotic oscillators. It has been shown that these motifs are prone to be phase-locked with the cell cycle as they are largely influenced by the effects of gene density due to genome replication events [60]. Over the course of the cell cycle, the cell volume increases steadily until cell division; however, the genome copy number doubles more or less instantaneously if there is only one genome copy per cell. In these cases, the simple negative transcriptional feedback loops are locked to the cell cycle causing them to lose their autonomous functionality as a biological timekeeper [60]. One very potent strategy to overcome this phase-locking is to increase the genome copy number normally found in cells. The circadian clock model organism *Synechococcus elongatus* PCC 7942, for example, naturally harbours four genome copies per cell. Increasing the natural copy number weakens the otherwise drastic effect of DNA replication events on the overall gene density, because normally not all genome copies are copied at the same time but rather one by one. This causes the gene density to gradually increase, which almost eliminates a phase-locking to the cell cycle [60].

**Figure 2.**
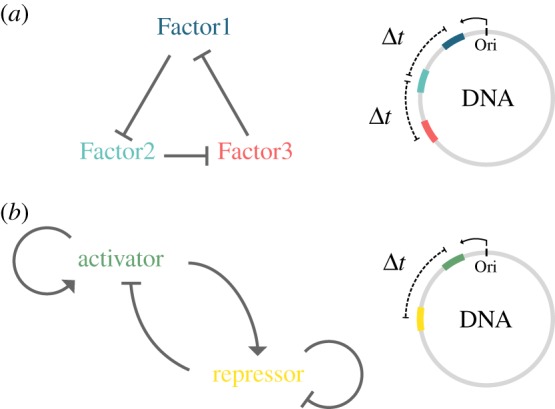
Representation of two synthetic oscillators and genome integration. (*a*) The network architecture of the repressilator by Elowitz & Leibler [61]: Factor1 represents a protein that represses the production of Factor2, which in turn represses the production of Factor3. Factor3 acts as a repressor for the gene expression of Factor1 again. The three factors are colour coded and their location on a circular chromosome is shown on the right. The Δ*t* represents the distance on the chromosome and thus the time difference between the replication of the genes. (*b*) The network architecture of the dual-feedback oscillator by Stricker *et al*. [62]: the activator activates its own production and enhances the production of the repressor. Whereas the repressor prevents its own production and suppresses the production of the activator. The two factors are colour coded and their location on a circular chromosome is shown on the right. The Δt represents the distance on the chromosome and thus the time difference between the replication of the genes. Ori depicts the origin of replication and the arrow indicates the direction of replication. This figure is adapted from [63] where the effects of the position on the chromosome of these two synthetic oscillators are studied in more detail.

This is also an important part to remember when building a synthetic oscillator. Usually, synthetic constructs are introduced to cells on plasmids, which are randomly replicated with 10–100 copies per cell, thus are not susceptible to phase-locking by the cell cycle. However, if you want to integrate your construct on the genome it is important to figure out the position on the genome as this might be essential for functionality due to the timing of replication. For two synthetic oscillators, the repressilator (figure 2*a*) [61] and the dual-feedback oscillator (figure 2*b*) [62], genome integration was analysed [63]. Depending on the structure of the synthetic oscillator different strategies arise regarding the position on the genome and the distance between each factor. The repressilator (figure 2*a*) [61] shows almost no locking when all three genes are close to each other and replicated at the same time. Whereas, when genes are placed further apart from each other on the genome locking to the cell cycle can be observed, increasing proportionally with the distance between genes. This effect does not disappear when noise is added to the timing of replication for each gene [63]. Interestingly, the opposite is true for the dual-feedback oscillator (figure 2*b*) [62]: strongest locking can be observed when genes are placed right next to each other on the chromosome, whereas locking diminishes when the distance between the two genes increases. However, locking never disappears as for the repressilator, even though noise has similar attenuating effects on the locking to the cell cycle in the dual-feedback oscillator [63].

As mentioned above, some cyanobacteria have found a way to make their circadian clock robust against the cell cycle by harbouring multiple genome copies per cell; however, they also have an additional way that helps prevent phase-locking to the cell cycle. By using a protein oscillator at the core of the circadian clock instead of nested TTFLs, cyanobacteria uncouple their timing machinery from the cell cycle. This is already sufficient to prevent phase-locking to the cell cycle; however, increasing the genome copy number is even better [60]. However, a PTO at the centre of a circadian clock alone is also susceptible to high protein decay or dilution rates. Thus, for cells dividing faster than 24 h, which show a high protein dilution, a TTFL in addition to a PTO is required to generate robust circadian rhythms [64,65].

Most cyanobacteria which have been shown to harbour a clock have in addition to the protein oscillator multiple genome copies; thus, their timing system is remarkably robust against the cell cycle. However, the picocyanobacteria *Prochlorococcus*, which have a reduced genome and circadian clock compared to *Synechococcus elongatus* PCC 7942, have only one genome copy per cell. In these cyanobacteria, we see a strong coupling between the circadian clock and the cell cycle as they stably divide once per 24 h with distinctly timed cell cycle phases [43,66].

In addition to the PTO at the core of the circadian clock, cyanobacteria use a second protein network to transfer the clock state to gene regulation, which further uncouples the clock from the cell cycle. This push–pull network with RpaA at its centre is complemented by SasA, CikA and RpaB depending on the cyanobacterial strain (figure 1) [16]. Interestingly, the precision with which circadian clocks tell time increases with the number of oscillations, i.e. genes involved in the readout of the clock [67]. Furthermore, cross-regulatory interactions of proteins in the readout of a clock, e.g. the RpaA network, can increase the transmission of temporal information [67].

We have seen that there are various strategies to uncouple the oscillator from the cell cycle and make it robust against changes in the protein and nucleotide levels. This can either be achieved by increasing the genome copy number, by placing the genes for the oscillator on plasmids with higher copy number, or by integrating one or even multiple PTOs into the circadian oscillator.

## 6. Summary

Circadian clocks are a prevalent system to coordinate gene expression of large proportions of the genome. The ability to anticipate recurring environmental changes provides a fitness advantage, which is harnessed by organisms of every group of life. However, circadian clocks are only a specialized form of timing mechanisms defined by three characteristics, i.e. entrainment, free running, temperature compensation, that produce oscillating gene expression. Regulating large parts of the genome in a timely fashion is an even more widespread phenomenon, which exhibits different forms besides circadian clocks that we did not cover in this review.

Over the last decade, different parts of the circadian clock in cyanobacteria were characterized through mathematical modelling gaining insights into circadian regulation that go beyond cyanobacteria allowing us to understand general features of circadian clocks. The understanding of the molecular mechanisms underlying this timing system in cyanobacteria including the ordered phosphorylation of the KaiC monomers [37], the interaction with KaiA and KaiB [31], as well as the influence on gene regulation [40] provided the basis for further studies. Through mathematical models, we have seen how this system can potentially robustly filter noise of the exogenous signal and maintain its inner rhythm [56]. Nevertheless, these models also showed us the limitations of this circadian clock when noise is too high or in an adverse moment [55]. Some of these models even though they devote themselves to the cyanobacterial circadian clock show us a universal way how to uncouple timing systems from the cell cycle making them robust against abruptly changing nucleotide concentrations [60,67]. The computer simulations by Troein *et al*. [47] helped to understand the environmental conditions that lead to the evolution of circadian clocks. However, these simulations took TTFLs as the basis for their analyses and did not consider PTOs as well. Nevertheless, their results can be considered universally applicable for the evolution of circadian clocks.

In the future, it will be interesting to see if the size of the cyanobacterial circadian clock can be reduced based on the molecular understanding of interactions of protein factors and still retain full functionality. In addition, the design principles unravelled by mathematical models and the biochemical characterization of protein domains provide the basis for further protein engineering and synthetic biology approaches to building a timing system and oscillator from scratch. These newly designed timing systems can then be used to artificially regulate gene expression of engineered metabolic pathways to coordinate the production of metabolites as efficiently as possible in a natural noisy environment. Furthermore, the understanding of how biological systems robustly cope with noise can help to shape the future of other research areas beyond biology, e.g. in computing architecture and new algorithms for parallel computing.

## Data accessibility

This article has no additional data.
